# Properties of Eco-Friendly Particleboards Bonded with Lignosulfonate-Urea-Formaldehyde Adhesives and pMDI as a Crosslinker

**DOI:** 10.3390/ma14174875

**Published:** 2021-08-27

**Authors:** Pavlo Bekhta, Gregory Noshchenko, Roman Réh, Lubos Kristak, Ján Sedliačik, Petar Antov, Radosław Mirski, Viktor Savov

**Affiliations:** 1 Department of Wood-Based Composites, Cellulose and Paper, Ukrainian National Forestry University, 79057 Lviv, Ukraine; 2 Department of Chemistry, Ukrainian National Forestry University, 79057 Lviv, Ukraine; noschenkog@gmail.com; 3 Faculty of Wood Sciences and Technology, Technical University in Zvolen, 960 01 Zvolen, Slovakia; reh@tuzvo.sk (R.R.); sedliacik@tuzvo.sk (J.S.); 4 Faculty of Forest Industry, University of Forestry, 1797 Sofia, Bulgaria; p.antov@ltu.bg (P.A.); victor_savov@ltu.bg (V.S.); 5 Department of Wood-Based Materials, Poznań University of Life Sciences, 60-627 Poznań, Poland; rmirski@up.poznan.pl

**Keywords:** magnesium lignosulfonate, sodium lignosulfonate, bio-based adhesives, urea formaldehyde resin, wood-based composites, particleboards, formaldehyde content, physical and mechanical properties, acid-base buffer

## Abstract

The purpose of this study was to evaluate the feasibility of using magnesium and sodium lignosulfonates (LS) in the production of particleboards, used pure and in mixtures with urea-formaldehyde (UF) resin. Polymeric 4,4′-diphenylmethane diisocyanate (pMDI) was used as a crosslinker. In order to evaluate the effect of gradual replacement of UF by magnesium lignosulfonate (MgLS) or sodium lignosulfonate (NaLS) on the physical and mechanical properties, boards were manufactured in the laboratory with LS content varying from 0% to 100%. The effect of LS on the pH of lignosulfonate-urea-formaldehyde (LS-UF) adhesive compositions was also investigated. It was found that LS can be effectively used to adjust the pH of uncured and cured LS-UF formulations. Particleboards bonded with LS-UF adhesive formulations, comprising up to 30% LS, exhibited similar properties when compared to boards bonded with UF adhesive. The replacement of UF by both LS types substantially deteriorated the water absorption and thickness swelling of boards. In general, NaLS-UF-bonded boards had a lower formaldehyde content (FC) than MgLS-UF and UF-bonded boards as control. It was observed that in the process of manufacturing boards using LS adhesives, increasing the proportion of pMDI in the adhesive composition can significantly improve the mechanical properties of the boards. Overall, the boards fabricated using pure UF adhesives exhibited much better mechanical properties than boards bonded with LS adhesives. Markedly, the boards based on LS adhesives were characterised by a much lower FC than the UF-bonded boards. In the LS-bonded boards, the FC is lower by 91.1% and 56.9%, respectively, compared to the UF-bonded boards. The boards bonded with LS and pMDI had a close-to-zero FC and reached the super E0 emission class (≤1.5 mg/100 g) that allows for defining the laboratory-manufactured particleboards as eco-friendly composites.

## 1. Introduction

Particleboards still predominate in the world production of wood-based composites. In 2018, industrial particleboard production reached a record output of 97 million m^3^ worldwide [1]. Particleboards remain one of the most important value-added panel products in the wood industry and are widely used in various fields of human activity. With increasing applications of composite materials, the demand for adhesive increases. The cost of resin is about 30–50% of the material costs, whereas the product contains only 2–14% of resin in terms of the amount associated with the dry weight of wood. Even at such low concentrations, the cost of the resin is the main factor significantly affecting the total product price [2].

Today, the main classes of thermosetting adhesives that have been dominating the field of wood composites industry for many decades are amino-based, phenolic, and isocyanate resins [3]. Currently, approximately 95% of the total number of adhesives used for the manufacture of wood composites are formaldehyde-based resins [4], and the most predominant type is urea-formaldehyde (UF) resins, the total consumption of which is estimated to approximately 11 million tons per year [5]. Within these thermosetting adhesives, UF resins are the most widely used adhesives in the manufacture of wood-based composites, including particleboards. The industrial success of these resins is associated with their low-cost raw materials, high reactivity, excellent adhesion to wood, ease of use for a wide range of curing conditions, low temperature of curing, short pressing time, aqueous solubility, and a colourless glue line [6]. However, they have a major drawback, connected to the hazardous emission of volatile organic compounds (VOCs) and free formaldehyde from the finished wood-based composites, which can irritate the eyes, respiratory, and nervous systems and even lead to cancers such as leukaemia [7]. As a result, new formaldehyde emission restrictions have been set for wood-based composites in Europe, the United States, and Japan. In addition, the production of these resins relied on non-renewable oil resources. Today, these adhesives are sufficient for supply, but the shortage of petroleum products may affect the future cost and availability of these petroleum-based adhesives. Therefore, there is a growing interest in the development of environmentally friendly adhesives for wood from renewable resources. This has stimulated the transition from traditional formaldehyde-based synthetic resins to new environmentally friendly adhesives for the production of eco-friendly wood-based panels from renewable resources. Lignin-based products are one of the most promising environmental alternatives to traditional formaldehyde resins [8,9].

The main interest in lignin is due to its phenolic structure with several favourable properties for the manufacture of wood adhesives, such as high hydrophobicity and low polydispersity [10]. However, the low chemical reactivity of lignins requires higher concentrations of catalysts (heat or acid) and longer heating times are required during the production of wood-based composites [9,11,12,13]. Hence, an additional chemical modification of lignin is required to increase the lignin reactivity to formaldehyde [13,14,15]. Peng and Riedl [16] proved in their work that the reactivity of lignosulfonate with formaldehyde increases when wheat starch is added as filler. It is estimated that the planet currently contains 3 × 10^11^ metric tons of lignin with an annual biosynthetic rate of approximately 2 × 10^10^ tons [13]. There are large quantities of technical lignin (mainly as kraft lignin and lignosulfonate) generated as waste or a by-product from the paper making industry, with an annual global production of approximately 50–75 million tons [17]. Thus, the utilisation of lignin as a renewable component in the production of value-added products, including wood adhesives [18,19,20,21], could be an efficient way to achieve sustainable resource management.

Many authors have extensively studied the potential utilisation of different types of lignin including lignosulfonates (LS), Kraft lignin, organosolv lignin, enzymatic hydrolysis lignin, and soda lignin in adhesive applications [12,15,18,20,21,22,23,24,25,26,27,28,29]. The drawbacks of using lignins alone as wood adhesives, modifications to enhance the reactivity of lignins, and production of lignin-based copolymer adhesives for composite wood panels are reviewed and discussed in the comprehensive review [30]. In several studies, lignin was used as the binding agent for oriented strand boards [31] and medium density fibreboards (MDF) [27,28,29], which allowed the production of eco-friendly, low-toxic boards. The main scientific and industrial interest in lignin-based wood adhesives, such as LS, is due to the polyphenolic structure of lignin, allowing a partial replacement of phenol in phenol-formaldehyde (PF) resins. Çetin and Özmen [32] demonstrated that organosolv lignin could be used to replace 20–30% of the phenol in PF resins used to bond particleboards, without adversely affecting bond properties. It was found the phenolated lignin exhibited better mechanical properties than the unmodified lignin. In another study [33], it was also attested the possibility of substitution of up to 30% of phenol by lignin in PF adhesive without significantly affecting the plywood shear strength. Akhtar et al. [34] found that the maximum shear strength and wood failure was obtained by 20% addition of LS to PF resin. da Silva et al. [35], using LS with pure and in mixtures with phenol-formaldehyde (PF) resin to produce particleboards, found that the replacement of PF adhesive by LS in up to 80% was satisfactory in meeting the specific standard for mechanical properties. However, it was not possible to produce boards with minimal strength properties with pure LS-based adhesive. In other works [36,37], particleboards bonded with glyoxalated lignin combined with pMDI showed superior internal bond (IB) strength in dry and boiled conditions. There are also a number of successful attempts to produce MDF in laboratory conditions on the basis of LS [8,21,38,39,40,41,42,43,44]. The authors showed that the use of LS as an adhesive is a perspective approach for producing eco-friendly MDF panels without harmful free-formaldehyde emissions. Consequently, various studies have been carried out with a number of different lignin types as substitutes for phenol in PF adhesives. However, to date, the addition of unmodified or modified lignin-based compounds into UF adhesives is limited.

Since recently, due to environmental trends, focus has been drawn to the use of LS in adhesive compositions for wood [38,39,41]; thus, it was of considerable interest to study the effect of LS on the pH of UF compositions. This is due to the fact that the acidity of UF compositions is one of the key factors determining their pot life, cure rate, cure temperature, depth of cure, cohesion and adhesion, moisture resistance and atmospheric influences resistance, and other properties [45,46,47]. Despite this, to date, the change in pH during the curing of UF resins with natural fillers has not been fully investigated. This information may also be helpful in understanding the effect of pH on the curing of pure UF resins.

Considering the above, the objective of this work was to evaluate the feasibility of using lignosulfonates as an adhesive and the effect of replacing the UF resin by different proportions of lignosulfonate on the physical and mechanical properties of particleboards. The effect of lignosulfonates on the pH of lignosulfonate-urea-formaldehyde adhesive compositions was also investigated.

## 2. Materials and Methods

### 2.1. Materials

**Figure 1:**
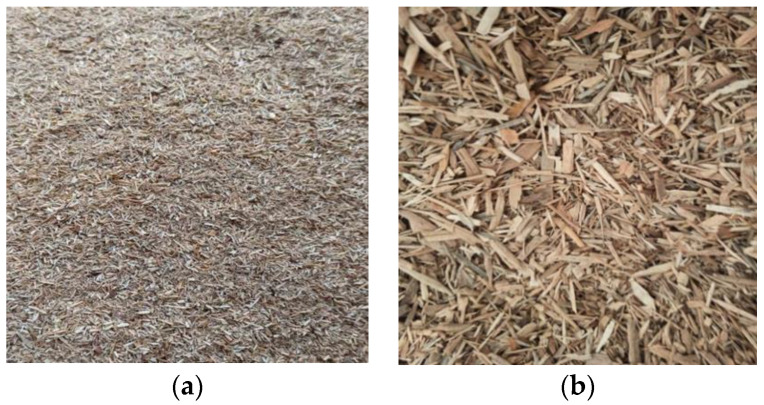
Appearance of the wood particles used for outer layers (**a**), core layer (**b**).

Factory-produced wood particles comprised of coniferous (75%) and deciduous (25%) species (origin—the Ukrainian Carpathians, Ivano-Frankivsk region) were obtained from the local particleboard plant. The moisture content of wood particles, determined by the drying-weighing method, was approximately 8%. The fractional composition of the particles for the outer and core layers of the boards is presented in Table 1, and the appearance of the particles is shown in Figure 1.

The lignosulfonate-urea-formaldehyde adhesive system (LS-UF) consisted of UF resin grade A (density 1.28 g/cm^3^, solid content 66%, Ford cup (4 mm, 20 °C) viscosity 98 s, pH = 8.05, gel time 50 s) (producer Karpatsmoly LLC, Kalush, Ukraine), pMDI resin Ongronat^®^ WO 2750 (NCO content—31.05 wt %, viscosity at 25 °C, 201 MPa·s, acidity as HCl—120 mg/kg), paraffin emulsion, urea, ammonium sulphate, magnesium lignosulfonate (Borregaard, Germany), and sodium lignosulfonate (Domsjö Lignin, Sweden). The water solution with 43% of ammonium sulphate [(NH_4_)_2_SO_4_] was used as hardener and mixed with the resin before spraying into wood particles. The water solution with 40% of urea and water solution with 50% of LS were mixed with the resin. Urea and LS were used as water solutions at 40% and 50% working concentrations, respectively. The lignosulfonate addition levels were based on the replacement of 10%, 20%, 30%, 50%, 75%, and 100% of the UF resin in the adhesive system used in outer and core layers. Magnesium lignosulfonate (MgLS) had the following characteristics: total solids content—min 90%; pH (10%)—4.0 ± 1.0; insoluble matter [%]—max. 0.8; Mg [%]—3; Cl [%]—≤0.1; sucrose [%]—6; density [kg/m^3^]—450–600. Sodium lignosulfonate (NaLS) had the following characteristics: total solids content > 95%; pH (10% solution)—6 ± 1; sodium Na—9; sulphur, S—8.5; calcium, Ca—0.12; chlorine, Cl—0.01; insoluble substances <0.1; sulphate—7.5 in the form of sulphate ions; sucrose—2.0.

In order to use lignin of LS as the main component in adhesive formulations, its low reactivity needs to be compensated by using a suitable crosslinker [15]. pMDI was used as an additional component to improve moisture resistance and mechanical properties for LS adhesives due to its high reactivity towards wood surface, LS molecules, and UF resin molecules. In order to find out how the addition of pMDI resin to the adhesive compositions affects the properties of the particleboards, the boards were made using UF and LS adhesives with different content (1%, 3% and 5%) of pMDI resin.

### 2.2. Measurement of pH

The pH was measured using pH-meter pH-301 and combined electrode with glass membrane (glass/KCl, AgCl/Ag). A two-point calibration procedure was performed with pH buffer solutions of pH 4.01 ± 0.01 and 6.86 ± 0.01 before and after pH measurements. To measure pH, 40 g samples were prepared by weighing the components with an accuracy of 0.001 g. Glass electrode was placed into the sample and then the sample was stirred for at least 5 min. The pH values were measured after stopping stirring. The stirring procedure and measurements were repeated three times, and the average pH value was calculated. When measuring pH, it was observed that to obtain stable pH values for lignosulfonate solutions, stirring for slightly longer periods of time was required compared to standard solutions. Measurement of pH values for each point during titration of lignosulfonates solutions was also performed after 5 min of stirring. To determine the effect of each component on the pH of adhesive composition, the components were added to UF resin one by one, thoroughly mixed, and the pH was measured. The components were mixed in the following sequence—UF resin, LS, urea, ammonium sulphate, paraffin emulsion. The content of UF resin and LS was changed in the compositions and the other components were added in the same amount: paraffin emulsion—7.1%; 33% ammonium sulphate solution—5.4%; 43% urea solution—4.3%.

### 2.3. Manufacturing of Particleboards

**Figure 2:**
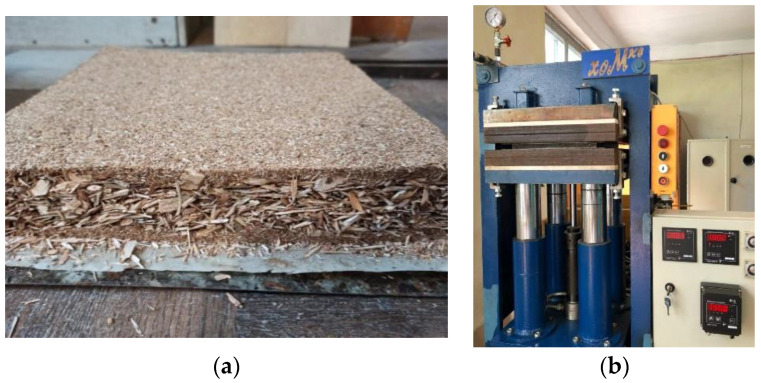
The formed particle mattress (**a**) and hydraulic laboratory press (**b**).

In this study, three-layered particleboards of 290 mm × 290 mm dimensions and a thickness of 16 mm with a target density of 650 kg/m^3^ were designed. The amount of UF resin, pMDI resin, urea, hardener, and paraffin emulsion that were needed for the blending process differed between core layer and outer layer. It is due to the temperature difference between surface and core caused by the heat transfer from the surface to the core of particleboard. In addition, different amounts of resin and additives used are due to the difference in surface area of particles used in outer and core of particleboard. In control series, the amount of solid UF resin was 14 wt % and 9 wt % based on the mass of oven dry wood particles for outer and core layers, respectively. During resin mixing, 2.3% and 0.5% of urea solution and 0.2% and 0.6% of ammonium sulphate based on dry particles weights were mixed with 14% and 9% of UF resin for outer and core layers, respectively. On the other hand, 0.8% of paraffin emulsion based on dry particles weight was also incorporated into the resin mixtures. pMDI resin was added to the adhesive system used for core layer. Wood particles were blended with the adhesive by hand. After blending, the resinated particles were hand spread evenly onto a 290 mm × 290 mm wooden box with a caul plate as the base to form the mattress (Figure 2a). The mattress formed was then pre-pressed manually to consolidate the thickness. Next, the mattress was subjected to hot pressing in an automatically controlled hydraulic laboratory press “xoMкo”(LLC “ODEK” Ukraine, Ukraine) (Figure 2b) at the pressure of 2.5 MPa, and temperature 200 °C for 600 s (during the last 30 s of the press cycle, the pressure was continuously reduced to 0 MPa). The high pressing temperature and extended pressing time are required for activating lignosulfonates and their binding to wood particles [8,15,30,39,41]. The long pressing time was also to make it possible to evaporate all the water present in the composition of the LS-UF adhesive. The experimental design for this study is summarised in Table 2.

### 2.4. Particleboards Testing

After pressing, boards were stabilised in air until reaching room temperature. Then, the boards were conditioned for one week in a conditioning room maintained at a relative humidity of 65 ± 5% and 20 ± 2 °C prior to properties evaluation. Three boards were produced for each type of particleboards in the experimental design, i.e., 39 boards in total +1 set of control. The control particleboard was made from adhesive system without LS. The conditioned boards were cut into required testing size according to relevant standards. Three samples of each board were tested according to the European standards for moisture content (EN 322) [48], density (EN 323) [49], bending strength (MOR) (EN 310) [50], modulus of elasticity (MOE) (EN 310) [50], internal bond (IB) (EN 319) [51], and thickness swelling (TS) (EN 317) [52]. On the other hand, for each series, one board was randomly selected for analysis of formaldehyde content (FC) based on EN ISO 12460-5 (perforator method) [53].

### 2.5. Statistical Analysis

The effects of LS content on the properties of the laboratory-fabricated boards were evaluated by analysis of variance (ANOVA) at the 0.05 level of significance. Duncan’s Range tests were conducted to determine significant differences between mean values.

## 3. Results

### 3.1. The Effect of Lignosulfonates on the pH of the Adhesive Compositions

It is known that the acid value—pH—is an important characteristic of UF adhesive compositions [45,46,47]. For long-term storage of resin in liquid state, a pH close to 7–8 is maintained in it. To cure it, acidic hardeners are added, which lower the pH. As a result of the decrease in pH, rapid condensation processes of UF oligomers begin, which leads to the curing of the composition [47]. In addition, curing is further accelerated by heating. To better understand the effect of pH, it is worth noting that when the pH of the composition changes by 1, the curing rate of UF resin changes by about 10 times [45]. Hence, it is important to investigate the effect of LS on the pH of adhesive compositions. Since 50% solutions of both LS were acidic, in particular, NaLS is characterised by pH = 5.44 and MgLS has pH = 3.56. At first glance it seems that their addition should accelerate the curing of the adhesive composition. However, the research indicates that the addition of LS can slightly reduce the effectiveness of latent hardeners such as (NH_4_)_2_SO_4_.

**Figure 3:**
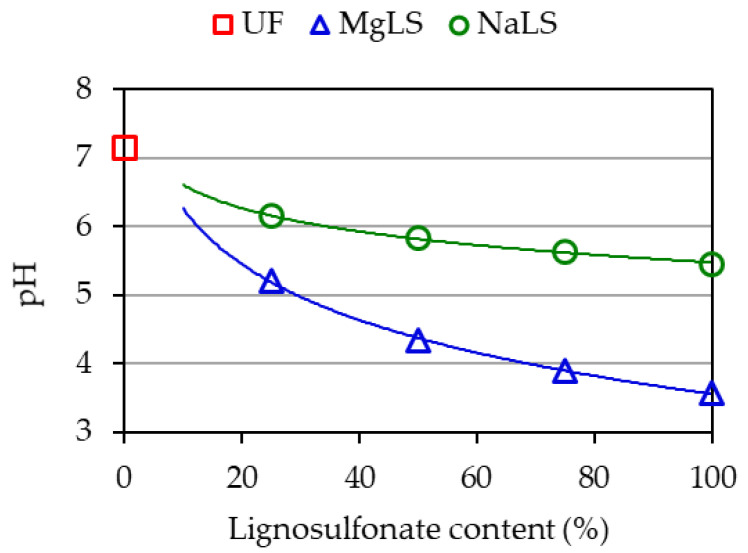
pH of freshly prepared uncured UF compositions as a function of lignosulfonate content.

The results of measuring the pH of individual components and uncured adhesive compositions are shown in Table 3, Table 4, Table 5 and Figure 3. The acidity of pure pMDI was not measured due to its incompatibility with aqueous solutions.

As is seen from Table 3, the UF resin had a slightly alkaline medium, pH = 7.615. The addition of NaLS significantly reduced the pH of the resin. It stands to mention that despite the acidic nature of LS, when they were added, no thickening and gelation of the UF composition was observed, which would interfere with further work. The addition of urea solution had almost no effect on the pH of the composition. The addition of hardener, (NH_4_)_2_SO_4_, also had little effect on the acidity of the compositions. The effect of adding (NH_4_)_2_SO_4_ was only noticeable for the pure UF resin not containing NaLS. A similar decrease in the pH of the uncured resin by 0.8 with the addition of NH_4_Cl has been noted [47]. The addition of paraffin emulsion slightly moved the pH of the composition towards the alkaline side. Thus, the NaLS had the most significant effect on the pH of adhesive compositions. Figure 3 shows that the effect of NaLS on the acidity of the original composition was proportional to the amount of added NaLS. From the data in Table 4, it is noticeable that the more NaLS is contained in the composition, the less the other components affect its acidity, regardless of whether they are acidic or alkaline in nature. This suggests that NaLS has acid-base buffering properties. Buffering properties mean the ability to resist a change in the pH of a solution when other substances of acidic or alkaline nature are added.

A similar effect was observed for uncured compositions with MgLS (Table 5). The biggest change in the pH of the composition occurred when MgLS was added. (NH_4_)_2_SO_4_ and paraffin emulsion had little effect on the pH. (NH_4_)_2_SO_4_ markedly changed the pH only in the absence of MgLS. That is, MgLS also exhibits buffering properties with regard to changes in the pH of the composition.

For compositions with a 50 UF/50 MgLS ratio, the effect of pMDI on pH was investigated. First, 3% pMDI was added to the composition containing UF, MgLS, urea, (NH_4_)_2_SO_4_, and paraffin emulsion, and thoroughly mixed using an electric stirrer. In this case, the pMDI was dispersed into droplets, which are well distinguished in transmitted light in an optical microscope at 10× magnification. The resulting compositions with pMDI had an average pH of 4.392, which is almost equal to the pH of such compositions without pMDI (4.386). From then on, it was assumed that pMDI does not affect the pH of the compositions, and therefore, it was not added to the compositions intended for the study of pH. In addition, no pMDI was added to avoid adhesion of pMDI to the glass electrode of the pH meter.

It is worth pointing out that pMDI has the potential to increase the pH of compositions when organic co-solvents are used along with water. This is due to the fact that during hydrolysis, the -N=C=O isocyanate groups are converted into -NH_2_ amino groups, which further neutralise acids [54]. However, pMDI, which remains in the aqueous dispersion in the form of individual droplets, cannot significantly affect pH, since under such conditions, the condensation reaction should prevail in pMDI rather than the hydrolysis reaction. In addition, the hydrolysis product, due to its hydrophobic nature, should remain dissolved in pMDI droplets and not significantly affect the pH of the composition.

**Figure 4:**
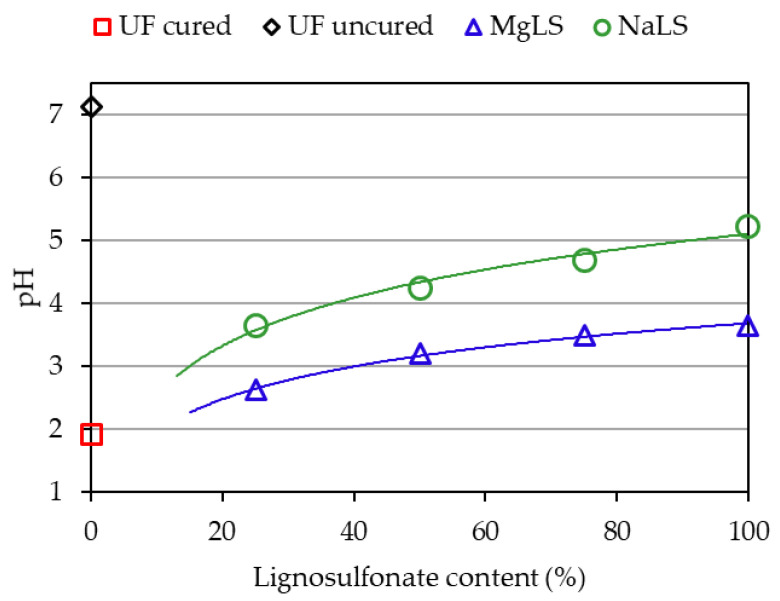
pH of cured UF compositions as a function of lignosulfonate content.

It also seemed important to investigate the effect of NaLS and MgLS on the pH of the compositions post curing and to what extent their buffering properties were exhibited in that case. However, pH measurement during the curing process is associated with technical challenges, in particular, with the adhesion of resin onto the electrode of the pH meter. Therefore, it was the pH of the cured compositions that was measured. First, the compositions consisting of UF, NaLS or MgLS, and (NH_4_)_2_SO_4_ were prepared. They were cured by heating to 98 °C for 15 min, cooled, left for 24 h, ground to a powdery condition, and the resulting powder was stirred in a double amount of water for 15 min. The pH of the resulting solution was measured. In doing so, a pH value close to that of the cured adhesive composition was obtained. The measurement results are shown in Figure 4. Paraffin emulsion and urea were not added, given that these components affect the pH insignificantly. In addition, this made it possible to see a clearer picture of the competition between lignosulfonates and (NH_4_)_2_SO_4_.

As is seen in Figure 4, the acid value of a composition consisting only of UF resin and (NH_4_)_2_SO_4_ post-curing decreased to pH = 1.914 (pH_uncured_−pH_cured_ = 5.228), which is consistent with the data of other authors [55]. On the other hand, the pH of compositions containing NaLS or MgLS changed far less. With an increase in the content of NaLS or MgLS, the pH of the compositions is close to the pH of pure NaLS or MgLS. The effect of NaLS or MgLS is most noticeable if we compare the pH of the compositions with a high content of lignosulfonates. In Figure 3 and Figure 4, it can be seen that in the uncured condition and post-curing, the pH value for them changes little (for example, for 75% NaLS pH_uncured_-pH_cured_ = 0.932, for 75% MgLS pH_uncured_-pH_cured_ = 0.407). Thus, NaLS and MgLS for cured adhesive compositions counteract pH change. Thus, NaLS and MgLS reduce the influence of the (NH_4_)_2_SO_4_ latent hardener.

It is known that a decrease in the acidity of UF-based adhesive compositions occurs due to the interaction of latent hardener ((NH_4_)_2_SO_4_) with resin formaldehyde according to the equation [45,47]:

2(NH_4_)_2_SO_4_ + 6CH_2_O = N_4_(CH_2_)_6_ + 6H_2_O +2H_2_SO_4_

In which case, a strong mineral acid H_2_SO_4_ is released, which reduces the pH of the medium to about 2. To simulate the interaction of the acid released by the latent hardener from lignosulfonates and to compare the effect of LNa and MgLS on pH, we studied the interaction in a system containing acid of known concentration, NaLS or MgLS of known concentration, but not containing UF resin. In parallel, to evaluate how the pH of a solution not containing NaLS or MgLS will change, an experiment adding acid to distilled water was conducted [56].

**Figure 5:**
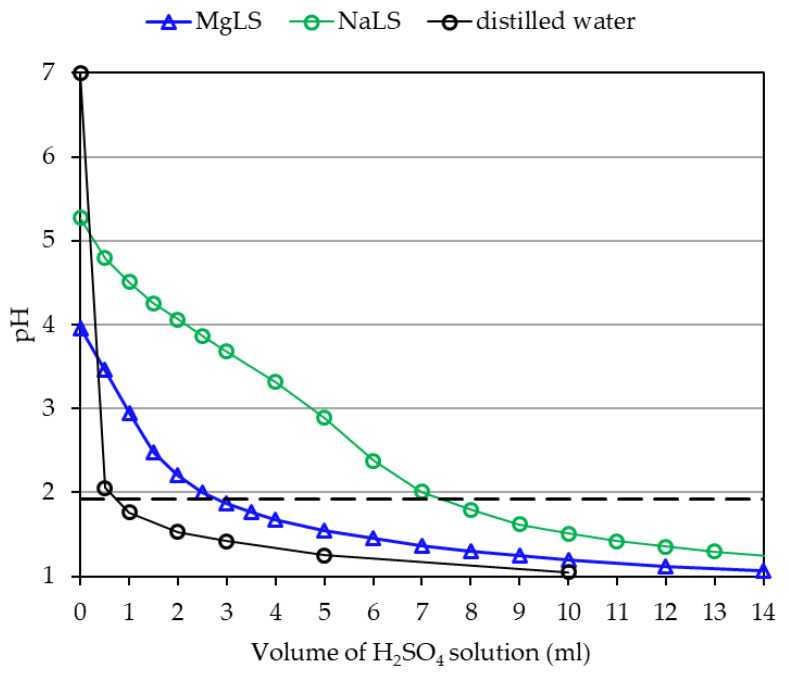
Curves of variation of pH as a function of titration with 0.309 M H_2_SO_4_ of lignosulfonate solutions or distilled water. Mass of the solution is 40 g. Concentration of lignosulfonate is 6%. The dotted line shows the pH of the cured resin with (NH_4_)_2_SO_4_ without lignosulfonates.

It should be noted that the effect of acid on the pH of water should be considered quite close to the effect of acid on the UF resin itself, because the resin contains almost no components that could counteract changes in pH. In Figure 5, it can be seen that the addition of 0.74 mL of acid enables reduction of the pH of water to 1.914 (change in pH = 7.000 − 1.914 = 5.086), but the pH of lignosulfonate solutions changes little with the addition of 0.74 mL of acid. For NaLS, the pH decreases only to 4.60 (change in pH = 5.270 − 4.60 = 0.67), and for MgLS, only to 3.20 (change in pH = 3.952 − 3.20 = 0.752). Thus, it is clearly seen from this graph that NaLS and MgLS exhibit buffering properties—that is, they resist pH changes. In this case, the buffer capacity of MgLS is greater than NaLS.

The LS neutralisation curve in Figure 5 can be useful for a rough estimation of the amount of acid or acid hardener that needs to be added to the composition to achieve the desired pH. For this, in Figure 5, we draw a horizontal line at pH = 1.914. This pH corresponds to curing the UF resin with (NH_4_)_2_SO_4_ without LS. Noticeably, achieving pH = 1.914 requires the addition of 0.74 mL of acid to distilled water. In the presence of 6% MgLS, achieving pH = 1.914 requires addition of 2.84 mL of acid. In the presence of 6% NaLS, achieving pH = 1.914 requires addition of 7.44 mL of acid. Thus, the presence of NaLS in the system requires an increase in the amount of acid by 7.44/2.84 = 2.6 times compared to MgLS. Similarly, an increase in the amount of a latent acid hardener will be required. If the need arises, a recalculation can be made for other contents of lignosulfonates. Of course, it should be remembered that other factors can affect the pH of the composition, too; this calculation is very rough, one might even say semi-quantitative.

The nature of the buffer properties of LS is explained by the presence in the LS molecules of (-COOM where M = Na^+^ or Mg^2+^) neutralised carboxyl groups and (-SO_3_M) sulfonic groups, the content of which, according to [57,58], can range within about 0.6–3.0 mmol/g and 1.5–2.0 mmol/g, respectively. These groups can bind H^+^ ions generated by the latent hardener according to the scheme [59]:

H_2_SO_4_ = 2H^+^ + SO_4_^2−^

Lignin-COO^−^ + H^+^ → Lignin-COOH

Lignin-SO_3_^−^ + H^+^ → Lignin-SO_3_H

The buffer properties of LS seem important because they can affect the properties of resins in the same way that buffering agents affect the properties of MUF resins [60].

Consequently, the addition of acidic NaLS and MgLS leads to a decrease in the pH of uncured UF resin. In contrast, when curing UF resin, LS counteract the effects of latent hardener ((NH_4_)_2_SO_4_) due to their acid-base buffering properties. As a result, the cured compositions have higher residual pH values. Thus, LS can be used to adjust the pH of uncured and cured LS-UF compositions.

### 3.2. Physical Properties of Particleboards

Table 6 presents the average values of density, TS, and water absorption (WA) after 2 and 24 h for the boards produced with the different adhesive compositions. The moisture content of the boards was within the range of 6%.

In general, it was observed that the LS type and its content in the adhesive system had a significant effect on the physical properties of the boards. The average values of the board densities were 618.5 kg/m^3^ and 626.6 kg/m^3^ for the boards bonded with the addition of MgLS and NaLS, respectively. This difference in density values is small and most likely caused by the conditions of manual mattress forming. No significant differences were observed between the boards bonded by MgLS or NaLS and UF adhesive system. Moreover, the average values obtained were somewhat inferior to the density calculated of 650 kg/m^3^ due to the effect of loss of materials during the mattress formation. The average density of reference boards (UF-bonded) was 624.5 kg/m^3^.

It was found that the replacement of the UF by both type of LS substantially influenced the TS after 2 h and 24 h, so that the higher the content of LS, the greater the TS. The same behaviour was observed in WA values after 2 h and 24 h. There was no significant difference in TS and WA values after 24 h between boards manufactured with UF adhesive (Ref) and boards manufactured with up to 20% replacement by MgLS (A and B). After 24 h, there was only no difference in TS values between UF adhesive (Ref) and 10% replacement by NaLS (G). That is, the replacement of up to 20% or 10% of UF adhesive by MgLS or NaLS, respectively, did not negatively alter the TS and WA values. The samples manufactured exclusively with 100% LS-based adhesives (F and L) after 2 h and after 24 h of immersion in water were delaminated. The samples manufactured with the replacement of 75% of UF by MgLS (E) and NaLS (K) were also delaminated after 24 h of immersion in water.

The boards produced with the conventional UF adhesive showed that the average thickness swelling values after 2 and 24 h of immersion in water were statistically lower in relation to the boards produced with the lignosulfonate-urea-formaldehyde adhesive. Significant differences were observed between the two types of lignosulfonates for the WA and TS after 2 and 24 h of immersion in water. The boards produced with the MgLS showed means statistically lower in relation to the boards produced with the NaLS; however, the differences in terms of absolute averages were small.

The high WA values in this study could be attributed to the low density of the boards and, accordingly, their high porosity. The low density of boards increased the boards’ water absorption for both 2 and 24 h. This result is in good agreement with the concepts mentioned by Maloney [2]. According to this author, the boards with higher density have better closure of its structure by reducing the permeability to water. For the thickness swelling, there was an increase in their average values for boards with higher specific mass. This increase results from the effects of the release of greater compression tensions of the boards produced with higher density. In addition, the higher values for WA and TS recorded in this study have been attributed to the fact that UF resins are characterised by their poor moisture resistance. This has been supported from literature stating that UF-bonded boards always have higher WA and TS than the corresponding PF-bonded board under the same experimental conditions [61].

The mean values of WA and TS after 2 and 24 h of immersion in water obtained in this study were also higher than the results reported by some researchers [62] in particleboards produced with lignin-phenol-formaldehyde resin with density of 750 and 950 kg/m^3^. In other works [38,40], it was also indicated that manufactured MDF panels using 15% gluing content of MgLS exhibited a deteriorated dimensional stability (thickness swelling and water absorption (24 h)). da Silva et al. [35] also observed that the replacement of PF resin by calcium and magnesium LS substantially influenced the WA and TS after 2 h and 24 h, so that the higher the proportion of LS, the greater the WA and TS. However, the replacement of up to 40% of PF by LS did not negatively alter the WA and TS values. Gothwal et al. [23] found that the replacement of phenol by lignin in PF adhesive in up to 15% did not alter the particleboard physical properties. On the contrary, Çetin and Özmen [32] observed that the TS and WA were largely unaffected by the presence of lignin in adhesives.

However, it should be noted that the dimensional stability of the boards bonded using LS-UF adhesive could be improved with the application of finishing materials for the board’s surface that assist in the waterproofing of the boards. Consequently, MgLS and NaLS, as lignin-based compounds, will require additional chemical modification (such as phenolation and methylolation, among others) in order to increase their chemical reactivity to formaldehyde and bonding efficiency [10,14,15,30].

**Figure 6:**
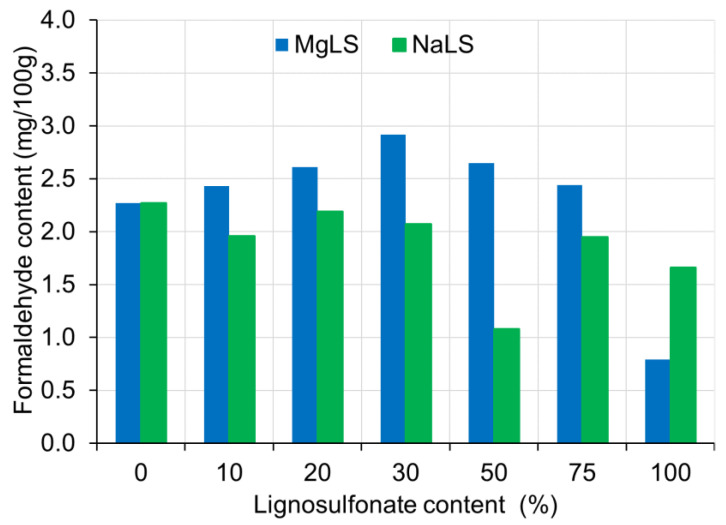
The formaldehyde content of boards made with lignosulfonated-urea-formaldehyde adhesive.

No clear dependence of formaldehyde content on the LS content in the LS-UF adhesive was found (Figure 6). In general, with the exception of pure adhesives, NaLS-UF-based boards had a lower formaldehyde content than MgLS-UF-based boards. This may be due to the higher pH of the cured compositions containing NaLS (Figure 4), since the influence of the residual acidity of the cured UF compositions on formaldehyde emission is known [55]. Of course, for UF resin compositions with LS, this dependence should be further researched. From the results, it can be also seen that NaLS-UF-based boards had a lower percentage of formaldehyde content than the UF-based boards used as control.

### 3.3. Mechanical Properties of Particleboards

**Figure 7:**
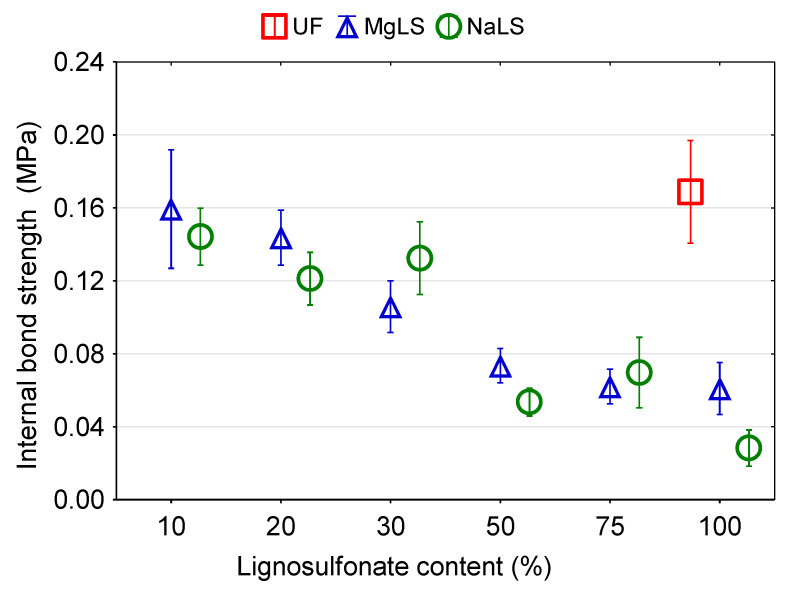
The internal bond (IB) strength of boards made with lignosulfonated-urea-formaldehyde adhesive.

**Figure 8:**
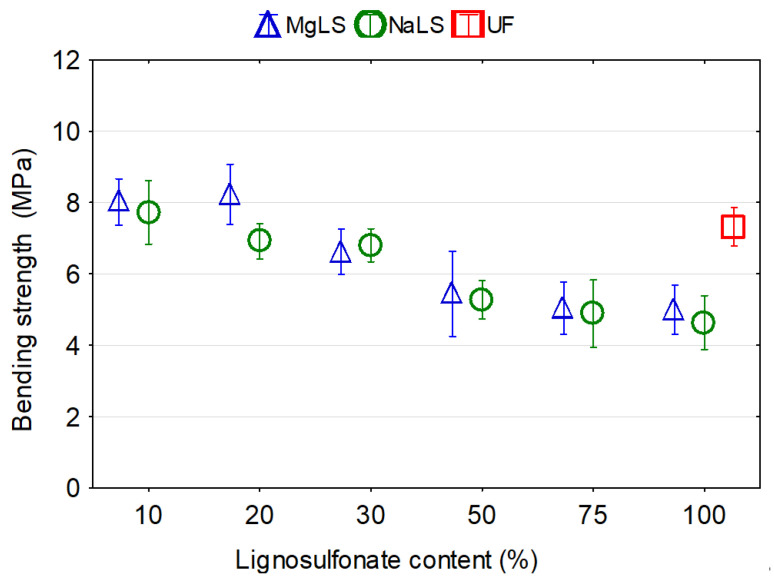
Bending strength (MOR) of boards made with lignosulfonated-urea-formaldehyde adhesive.

**Figure 9:**
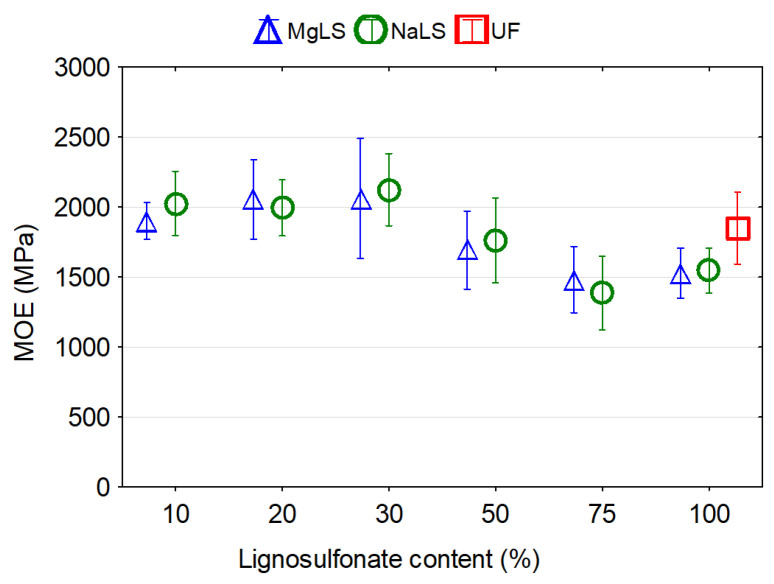
Modulus of elasticity (MOE) of boards made with lignosulfonated-urea-formaldehyde adhesive.

Graphical representation of the effects of the type and content of LS on the mechanical properties of particleboards are presented in Figure 7, Figure 8 and Figure 9. In general, it was observed that the LS type and content in the adhesion system had a significant effect on the mechanical properties of the boards.

The measured IB values of boards bonded using with LS-UF adhesives are summarised and compared with those of boards bonded UF adhesive in Figure 7. Typically, for both LS used in this work, IB tended to decrease with increasing the LS content. The IB of boards bonded with UF adhesive was significantly (*p* < 0.05) higher than that of boards bonded with LS-UF adhesives. Particleboards bonded with LS-UF adhesives with 10%, 20%, 30%, 50%, 75%, and 100% replacement of UF resin by MgLS showed reductions of 5.9%, 17.6%, 35.3%, 58.8%, 64.7%, and 64.7%, respectively, in the IB values compared to particleboards bonded with UF adhesive. A similar trend was observed in the reductions (17.6%, 29.4%, 23.5%, 70.6%, 58.8%, and 82.3%) in the IB values of boards bonded with 10%, 20%, 30%, 50%, 75%, and 100% replacement of UF resin by NaLS. To note, no significant differences were observed between the IB values for boards bonded with MgLS and NaLS. It can be concluded that there is an apparent correlation between IB and TS; the better a particleboard is bonded, the lower the thickness swelling. This is in a good agreement with similar findings of other researchers [41].

The incorporation of LS to UF resin formulation resulted in a reduction of bonding strength, because unmodified lignin has low reactivity toward formaldehyde [63]. Hence, lignin requires either chemical modification or molecular fractionation to improve its reactivity in the production of UF resins [20]. For example, the chemical reactivity of demethylated lignins was enhanced through diminishing methoxyl group content while increasing hydroxyl group content. With the lignins demethylated under optimum reaction conditions, the bonding strength of lignin-based phenolic resins increased while the formaldehyde emissions decreased [64,65,66].

Da Silva et al. [35] found that panels with pure PF and 20% replacement by calcium and magnesium LS had the highest average IB values of particleboards. As the PF adhesive was replaced by lignosulfonate in larger proportions, there was a decrease in the values of IB. A similar result was obtained by Akhtar et al. [34], who indicated that the maximum shear strength was obtained by 20% addition of lignosulfonate to PF resin. Çetin and Özmen [32] demonstrated that organosolv lignin could be used to replace 20–30% of the phenol in PF resins used to bond particleboards, without adversely affecting bond properties. Savov et al. [67] reported that MDF panels bonded with different lignosulfonate contents (20%, 30%, 40%) have also met the respective European standard requirements for applications in dry conditions.

The MOR and MOE data of the particleboards made using LS-UF adhesives are illustrated in Figure 8 and Figure 9.

Statistically significant differences were observed in mean values of MOR for the boards produced with UF and LS-UF adhesives. The replacement of 10–20% or 10% of the UF resin by MgLS and NaLS, respectively, caused positive changes in the MOR values by 9.4% and 12.4% for MgLS and by 5.6% for NaLS. From 30% or 20% replacement of UF resin by MgLS and NaLS, respectively, the MOR values decreased. Particleboards bonded with LS-UF adhesives with 30%, 50%, 75%, and 100% replacement of UF resin by MgLS showed reductions of 9.6%, 25.7%, 31.1%, and 31.7%, respectively, in the MOR values compared to particleboards bonded with UF adhesive. This can be explained by the greater fragility of the boards when increasing the content of lignosulfonate and a higher content of the steam–gas mixture in the process of pressing, associated with the increase in moisture content with an increased content of lignosulfonate solution [42,44]. Particleboards bonded with LS-UF adhesives with 20%, 30%, 50%, 75%, and 100% replacement of UF resin by NaLS showed reductions of 5.6%, 7.1%, 27.7%, 33.3% and 36.6%, respectively, in the MOR values compared to particleboards bonded with UF adhesive. There was no significance difference between MOR values for boards bonded with MgLS and NaLS. For both LS, it was evident that as the LS content increased, MOR tended to decrease. Savov and Antov [8] also observed a deterioration in the strength properties of the MDF panels at LS concentrations above 35%. The authors explained such deterioration of the strength properties by increasing the hot-pressing temperature. Antov et al. [42] reported that the increase in LS addition, from 10% to 15%, resulted in lower bending strength of the MDF panels. Quite similar results have been reported by Savov and Mihajlova [44], when they investigated the mechanical properties of MDF bonded with 5% UF resin and calcium LS (0% to 20% addition levels). In another work [35], it was found that the replacement of up to 60% of the PF adhesive by calcium and magnesium LS did not cause a negative change in the MOR values of particleboards. However, from 80% replacement of PF by LS and in boards produced with pure lignosulfonate, the MOR values decrease.

The MOR values found in this work were substantially lower in comparison with the mentioned by other authors [35]. This can be explained by the lower density of the boards and using UF adhesives with worse bonding properties in comparison with PF adhesives. It has been reported that low-density boards have low mechanical properties in general [68]. This might be also attributed to the presence of more sugars in the LS and increased moisture content of the pressed mat material, and higher vapour–gas mixtures at the higher LS addition levels [42].

There was no significance difference between MOE values for boards bonded with UF and LS-UF adhesives. The replacement of up to 30% of the UF resin by LS caused an increase in the MOE values up to 11.44% or 14.82% for MgLS and NaLS, respectively. However, particleboards bonded with LS-UF adhesives with 50%, 75%, and 100% replacement of UF resin by MgLS or NaLS showed reductions of 8.5%, 19.9%, 17.4% and 4.71%, 25.06%, and 16.44%, respectively, in the MOE values compared to particleboards bonded with UF adhesive. Savov and Mihajlova [44] also observed degradation, respectively decreasing, of bending strength and modulus of elasticity in bending of MDF panels but already after passing the calcium lignosulfonate content of 10%. Kouisni et al. [33] attested to the possibility of substitution of up to 30% of phenol by lignin in PF adhesive without significantly affecting the particleboard mechanical properties.

### 3.4. Effect of pMDI Content on the Properties of Particleboards

**Figure 10:**
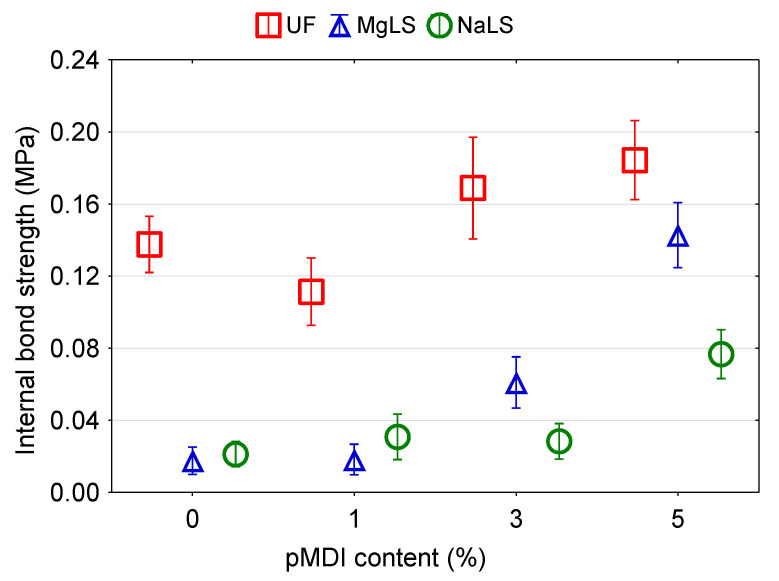
Effect of pMDI content on the IB of particleboards bonded with pure UF and lignosulfonate adhesives.

**Figure 11:**
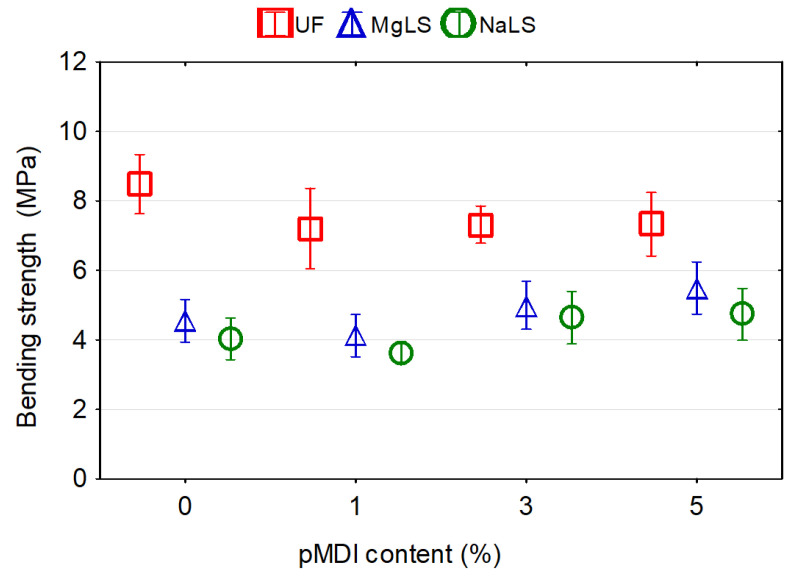
Effect of pMDI content on the bending strength of particleboards bonded with pure UF and lignosulfonate adhesives.

**Figure 12:**
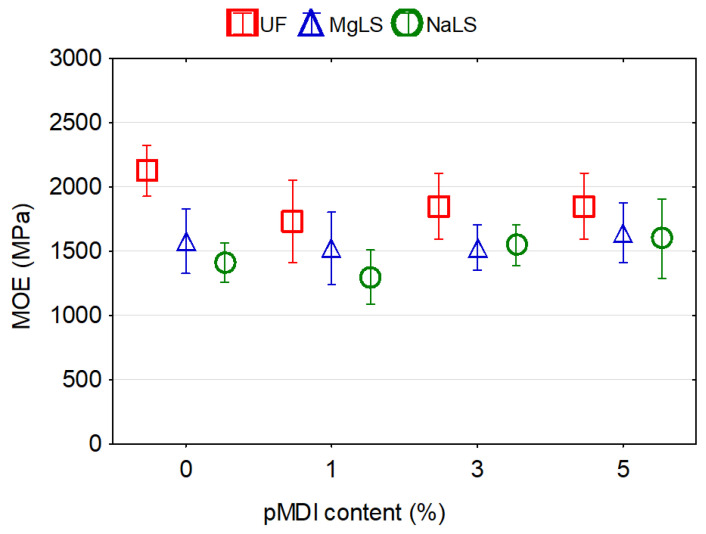
Effect of pMDI content on the MOE of particleboards bonded with pure UF and lignosulfonate adhesives.

**Figure 13:**
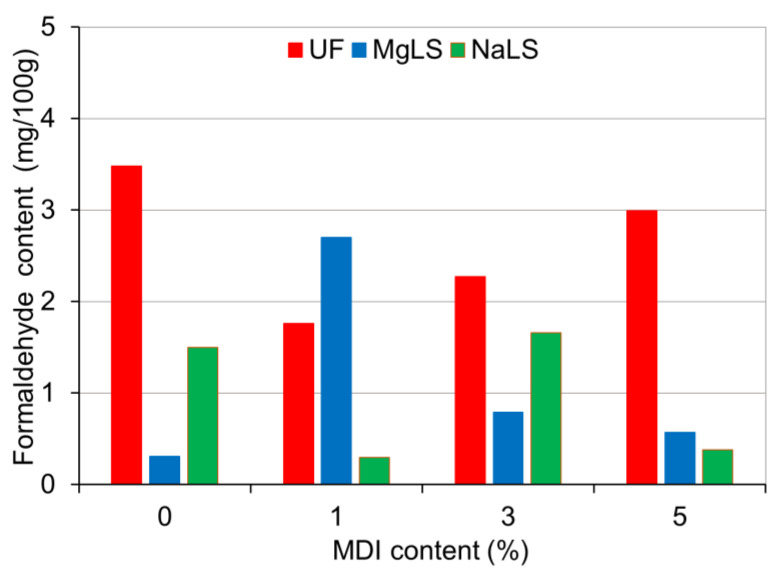
Effect of pMDI content on the formaldehyde content of boards made with pure UF and lignosulfonate adhesives.

It was observed that in the process of manufacturing boards using UF adhesive, increasing the proportion of pMDI resin in the adhesive composition from 1% to 5% increased by 50% the IB of the boards (Figure 10), did not affect the MOR (Figure 11) and at the same time decreased by 25% MOE (Figure 12). The formaldehyde content in the boards increased (Figure 13), although the average values of FC at pMDI content of 1%, 3%, and 5% are lower (1.76, 2.27, and 2.99 mg/100 g, respectively) compared to the boards made with UF adhesive without addition of pMDI resin (3.48 mg/100 g).

In the boards made using MgLS as an adhesive, the addition of pMDI resin increased MOR by 33.2% (Figure 11), MOE by 25.6% (Figure 12), and IB by 366.7% (Figure 10). The high IB strength was probably due to the chemical reaction of LS with pMDI in the cured adhesives, i.e., the formation of urethane linkages [15]. The effect of pMDI resin on the formaldehyde content in MgLS bonded boards is ambiguous (Figure 13). The lowest formaldehyde content was observed in the boards without the addition of pMDI resin (0.31 mg/100 g). Addition of 1% pMDI resin to MgLS adhesive significantly increased the formaldehyde content (2.70 mg/100 g), but a further increase in the pMDI resin content to 3% and 5% leads to a decrease in formaldehyde content (0.79 mg/100 g and 0.57 mg/100 g, respectively).

In the boards made using NaLS adhesive, the addition of pMDI resin can increase MOR by 35.7% (Figure 11), MOE by 17.9% (Figure 12), and IB by 133.3% (Figure 10). The effect of pMDI resin on the formaldehyde content in the NaLS bonded boards is also ambiguous (Figure 13). The lowest formaldehyde content was observed in the boards with a pMDI resin content of 1% (0.30 mg/100 g). Addition of 3% pMDI resin to NaLS adhesive significantly increases the formaldehyde content (1.66 mg/100 g), but a further increase in the pMDI resin content to 5% leads to a decrease in formaldehyde content (0.38 mg/100 g), reaching the super E0 class (≤1.5 mg/100 g). The determined formaldehyde emission value, which is much lower than 2 mg/100 g, i.e., equivalent to formaldehyde emission of natural wood [14], allows for the defining of manufactured particleboards as eco-friendly composites. The reference board, manufactured bonded with UF adhesive, can be classified under the E1 emission grade (≤8 mg/100 g). The results achieved in this study are in accordance with the results obtained by other several authors, where using different LS types in adhesive formulations for wood-based panels resulted in remarkably low formaldehyde content of the finished composites [9,38,40,42]. This might be attributed to the high amount of reactive groups in LS, which increase its reactivity towards formaldehyde [42,69].

In terms of mechanical properties, the boards based on UF adhesive with the addition of pMDI resin have higher values of MOR, MOE, and IB than boards based on LS adhesives with the addition of pMDI resin. In general, it can be stated that the addition of pMDI resin has a positive effect on the mechanical properties of particleboards. If we compare the properties of boards made using pure UF and LS adhesives (without adding pMDI resin), then here, the boards based on UF adhesive have much better mechanical properties than boards bonded with LS adhesives. However, the boards based on LS adhesives are characterised by a much lower formaldehyde content than the UF-bonded boards. In the boards based on MgLS and NaLS adhesives, the formaldehyde content is lower by 91.1% and 56.9%, respectively, compared to the UF bonded boards.

The previous studies [36,37] on the performance of particleboards bonded with glyoxalated lignin combined with pMDI also showed superior internal bond strength. This can be explained by the fact that the isocyanate groups in pMDI are highly unsaturated and can react with a number of active hydroxyl groups in wood particles’ surface and LS molecules, as well as with the moisture contained in the particles and the adhesive system [15]. Younesi-Kordkheili et al. [70] reported that addition of pMDI intensely improved the performance of lignin-urea-formaldehyde (LUF) resins and imparted a positive effect on the formaldehyde emission and water absorption of the panels. An additional small amount of pMDI (2%) was sufficient to yield panels with significantly greater bonding strength. In the work [71], it was shown that the introduction of pMDI to UF resin resulted in an improvement of bending strength and internal bond and in a reduction in formaldehyde content in the boards by as much as 30%.

## 4. Conclusions

Different adhesive systems were prepared by gradual replacement of UF-resin by magnesium or sodium lignosulfonates. MgLS and NaLS in LS-UF adhesive compositions exhibited acid-base buffer properties, expressed as resistance to pH change with the addition of acids. Compared to ammonium sulphate, NaLS and MgLS decrease the pH of uncured compositions and increase the pH of cured compositions. Lignosulfonates partially neutralise the effect of ammonium sulphate on the pH of cured compositions.

The results obtained demonstrate that when the content of MgLS or NaLS is associated with UF resin with 10–30% replacement by LS, the physical and mechanical properties are comparable with those of the UF-bonded particleboards. There was no significance difference between MOR values for boards bonded with MgLS and NaLS. It was not possible to produce particleboards with satisfactory physical and mechanical properties with pure MgLS and NaLS adhesives. The increased addition level of LS to UF resin formulation showed a reduction in bonding strength. Moreover, all manufactured particleboards bonded with LS-UF adhesives demonstrated significantly deteriorated moisture-related properties, such as WA and TS.

This study demonstrated the potential to combine LS and pMDI in particleboard manufacturing. The formaldehyde content of the particleboards bonded with pure MgLS and NaLS adhesives with the addition of pMDI, tested in accordance with the perforator method, was remarkably low (≤1.5 mg/100 g) and significantly different with the value of the reference UF-bonded boards, which allows for their classification as eco-friendly wood-based composites for indoor applications.

## Figures and Tables

**Table 1:** Fraction analysis (by % weight) of particles.

Outer Layer		Core Layer	
Screen Hole Size (mm)	Content (%)	Screen Hole Size (mm)	Content (%)
1.25	8.8	5.0	12.0
1.0	1.2	3.15	25.6
0.8	9.4	2.0	31.4
0.63	12.2	1.25	10.6
0.4	26.4	0.63	8.4
0.2	17.6	0.32	1.4
Dust	14.5	Dust	0.6
Total	100	Total	100

**Table 2:** Manufacturing parameters of particleboards produced in this work.

Board Type	Adhesive Type	UF Resin Content (%)	MgLS Content (%)	NaLS Content (%)
A	MgLS-UF	90	10	0
B	MgLS-UF	80	20	0
C	MgLS-UF	70	30	0
D	MgLS-UF	50	50	0
E	MgLS-UF	25	75	0
F	MgLS	0	100	0
G	NaLS-UF	90	0	10
H	NaLS-UF	80	0	20
I	NaLS-UF	70	0	30
J	NaLS-UF	50	0	50
K	NaLS-UF	25	0	75
L	NaLS	0	0	100
Ref		100	0	0

**Table 3:** pH values of the individual components of the adhesive compositions.

Component	pH
MgLS, 50% aqueous solution	3.565 ± 0.05
NaLS, 50% aqueous solution	5.442 ± 0.05
Paraffin emulsion 7.1%	9.414 ± 0.03
Urea, 1.8% aqueous solution	7.121 ± 0.05
(NH_4_)_2_SO_4_, 1.8% aqueous solution	5.682 ± 0.04
UF resin	7.615 ± 0.04

**Table 4:** pH values of freshly prepared uncured adhesive compositions of NaLS.

Mass Composition of the Mixture NaLS + UF Resin	pH Values of Uncured Adhesive Compositions			
	NaLS + UF	NaLS + UF + Urea	NaLS + UF + Urea + (NH_4_)_2_SO_4_	NaLS + UF + Urea + (NH_4_)_2_SO_4_ + Paraffin Emulsion
100% NaLS + 0% UF	5.442 ± 0.05	5.454 ± 0.05	5.459 ± 0.05	5.474 ± 0.04
75% NaLS + 25% UF	5.661 ± 0.04	5.667 ± 0.04	5.631 ± 0.05	5.652 ± 0.04
50% NaLS + 50% UF	5.867 ± 0.05	5.864 ± 0.05	5.837 ± 0.04	5.863 ± 0.05
25% NaLS + 75% UF	6.130 ± 0.04	6.146 ± 0.04	6.149 ± 0.04	6.197 ± 0.05
0% NaLS + 100% UF	7.619 ± 0.03	7.508 ± 0.03	7.142 ± 0.03	7.368 ± 0.04

**Table 5:** pH values of freshly prepared uncured adhesive compositions of MgLS.

Mass Composition of the Mixture MgLS + UF Resin	pH Values of Uncured Adhesive Compositions			
	MgLS + UF	MgLS + UF + Urea	MgLS + UF + Urea + (NH_4_)_2_SO_4_	MgLS + UF + Urea + (NH_4_)_2_SO_4_ + Paraffin Emulsion
100% MgLS + 0% UF	3.565 ± 0.04	3.569 ± 0.03	3.572 ± 0.03	3.589 ± 0.04
75% MgLS + 25% UF	3.837 ± 0.04	3.869 ± 0.03	3.897 ± 0.03	3.921 ± 0.04
50% MgLS + 50% UF	4.322 ± 0.03	4.334 ± 0.04	4.346 ± 0.03	4.386 ± 0.04
25% MgLS + 75% UF	5.140 ± 0.05	5.161 ± 0.03	5.203 ± 0.03	5.253 ± 0.04
0% MgLS + 100% UF	7.619 ± 0.03	7.508 ± 0.03	7.142 ± 0.04	7.368 ± 0.03

**Table 6:** Physical properties of particleboards produced in this work.

Board Type	Density (kg/m^3^)	Water Absorption (2 h) (%)	Water Absorption (24 h) (%)	Thickness Swelling (2 h) (%)	Thickness Swelling (24 h) (%)
Boards bonded with MgLS					
A	625.9 ± 44.1 bc	37.68 ± 3.93 b	47.55 ± 2.63 a	24.92 ± 4.80 b	40.85 ± 6.90 a
B	629.8 ± 44.3 c ^1^	37.46 ± 3.22 b	49.35 ± 1.48 ab	23.12 ± 4.73 b	43.85 ± 5.36 a
C	615.0 ± 29.1 abc	42.89 ± 3.43 c	51.50 ± 1.35 c	29.94 ± 5.27 c	52.37 ± 6.85 b
D	623.4 ± 39.9 bc	47.90 ± 1.18 d	58.46 ± 0.76 d	48.46 ± 6.07 d	80.92 ± 7.44 c
E	610.8 ± 37.1 ab	51.37 ± 4.53 e	Samples delaminated	51.69 ± 8.63 d	Samples delaminated
F	606.1 ± 26.8 a	Samples delaminated	Samples delaminated	Samples delaminated	Samples delaminated
Ref	624.5 ± 32.1 bc	31.43 ± 4.68 a	48.43 ± 3.39 a	17.71 ± 2.79 a	40.92 ± 3.56 a
Boards bonded with NaLS					
G	633.4 ± 38.6 bc	42.51 ± 5.33 b	50.48 ± 1.73 b	26.41 ± 2.98 b	44.73 ± 3.97 a
H	624.2 ± 44.3 abc	44.36 ± 4.65 b	55.55 ± 1.56 c	29.71 ± 4.63 b	49.88 ± 4.81 b
I	644.1 ± 42.9 d	44.36 ± 4.59 b	54.77 ± 2.38 c	32.25 ± 6.02 cb	59.08 ± 8.25 c
J	623.6 ± 35.6 ab	49.11 ± 1.95	56.32 ± 1.56 c	53.17 ± 7.66 d	87.23 ± 7.50 d
K	605.5 ± 52.0 a	50.13 ± 2.07 c	Samples delaminated	72.43 ± 12.61 e	Samples delaminated
L	629.5 ± 43.0 bc	Samples delaminated	Samples delaminated	Samples delaminated	Samples delaminated
Ref	624.5 ± 32.1 abc	31.43 ± 4.68 a	48.43 ± 3.39 a	17.71 ± 2.79 a	40.92 ± 3.56 a

^1^ Averages followed by the same letter at the column are statistically equal by the Duncan test at 95% probability.

## Data Availability

The data that support the findings of this study are available upon reasonable request from the authors.
